# The Cholera in Europe

**Published:** 1854-07

**Authors:** H. A. Johnson

**Affiliations:** Chicago


ART. III.—*The Cholera in Europe.* Read before the Cook County Medical Society, by H. A. Johnson, M.D.

At the last meeting of this Society, I had the honor to be appointed to prepare a report on the present views entertained by European physicians in regard to the Asiatic Cholera. In fulfilling this duty we have examined the journals, both foreign and domestic, so far as they were at my command.

The following extracts from Ranking's Abstract, Jan., 1854, contains all up to that time:

' Not much new light has been shed upon the immediate cause of cholera. The only novel suggestion is that of Dr. Snow,—that the disease is propagated by germs contained in the cholera evacuations, which germs are distributed through the air and in the water which is used for household purposes, in consequence of the want of proper attention to cleanliness. The swarms of flies which have been noticed at Newcastle during the present epidemic, and which were noticed in the epidemic of 1832 at Montreal, by Dr. Moore, and about the same time in a village near Edinburgh, by Dr. Knox may possibly have something to do with the matter. They seem, at least, to mark on phase of that blight in vegetation and murrain smong cattle, which has preceded the cholera scourge, and which still attends upon it. The fact is curious under any circumstances. The account of the swarms at Newcastle is by an anonymous writer to *The Lancet*, who states that he is not a member of the medical profession The cholera was at its height in the time referred to—September 21, 1853. The writer proceeds:—

The air in certain parts of the town is literally filled with a small fly with large wings, slightly tinged with green. I especially instance a space extending from the top of Northumberland Street in this town, to the town moor, and even across the moor The numbers of this fly, were, I found, greater over a sort of open drain at the end of the moor, which is crossed by the road more than half a mile from the town, there being merely a dwarf parapet to prevent any one falling in. In fact, this fly appeared to me to be spread generally over the district, and where the town commences, there the insects are more confined in space, and crowded in a dense cloud, and therefore more apparent.

I am not at all an alarmist, but I never in all my life saw anything like the quantity of this fly which I saw this afternoon when I went out for a walk before dinner. It was not a mere cluster of little clouds which could each be encircled in a hat, but a dense column, extending in space beyond the power of vision to discover where it commenced or where it ended I palpably walked into it and out of it as I have done in and out of a London fog. I shall never forget it. What is to be expected from the death and decomposition of this mass of insect matter?

The clearer notions about the local origin of cholera, and of the intimate relations subsisting between the disease and the various forms of fever, has resulted in clearer notions upon the subject of *prevention* These have shown sufficiently that it is in ventilation, in drainage, in personal cleanliness, and in other measures of the same kind, and not in quarantine, that any rational hopes of safety must rest. Dr. Gavin Milroy, however, has collected some additional facts in proof of the futility of quarantine."

He goes on to show that all attempts to prevent the spread of the disease by regulations restricting intercourse between affected and non-affected localities has been of no avail. It has steadily pursued its march from its native jungles till it has overspread the world.

Dr. Fuller, in the *Medical Times and Gazette*, for October, 1853, says, in reference to sulph. acid in the treatment of cholera:

"My own conviction is, that in sulph acid we have an antidote —a specific—against choleraic di rrhœa, if not against the worse forms of cholera, as powerful, as energetic, and as certain in its effect, as in cinchona bark or quinia, against a paroxysm of ague. In bilious diarrhœa, and in certain chronic diarrhœas, it is of little or no avail; and, in some, though in very few, according to my experience, it gives rise to pinching pain in th abdomen; but in epidemic diarrhœa, in accute autumnal diarrhœa, if I may so term it, and in more decided choleraic diarrhœa, I have known no single instance of its failure; indeed, the more choleraic the diarrhœa the more speedily are its curative effects produced This fact will appear of greater value when it is known that I have notes of its administration in above ninety such cases, many of which have occurred among my own patients at the hospital or elsewhere, and some in the practice of my friends.

With this howeʌer, even more than with other remedies success depends upon the mode of administration. Given at intervals of three or four hours, it fai s in exerting any curative influence in severe cases, and is seldom of much avail, even in the milder cases; and to insure its beneficial eff cts, it should be exhibited in full doses, repeated at much shorter intervals. When I first began to give it, I ordered two doses to be taken within the first hour, and a dose to be repeated every hour afterwards But recent and more extended exp ri nce, tog ther with the close and eager observation of its influence on my own person, has convinced me that, even in ordinary cases, twenty minutes form a sufficient interval between each dose. In severe cases, five or six doses may be given with advantage within the hour; and should any dose be rejected by the stomach, another should be administered at once. Rarely, however, is any dose rejected except the first, and the rejection of that is by no means common. While loathing the sights of other liquids, the patient, after he has tasted the first dose of the medicine, very generally craves for its repetition, and drinks it with avidity

As the stomach is very irritable, and the acid taken in simple water is, under circumstances, extremely palpable, I never mix it with syrup or other matters; and I strongly suspect the warm aromatic spices with which some of my friends have flavored it, are additions by no means agreeable to the patient. Unfortunately, I have had some porsonal experience in this matter, and so strongly was my distate to the medicine excited by such flavorings, that for the futnre whenever I am the patient, I shall certainly put my veto upon them.

Dr. Fuller gives the dilute acid of the pharmacopœa in ℨss. doses every 20 minutes till the discharge ceases.

The experiment of injecting a fluid into the veins has been repeated both in England and France. Dr Owen Rees makes the following judicious suggestions in reference to the constitution of the fluid and the conditions to be observed:

"With regard to the chemical constitution of the fluid, it would appear that we can scarcely venture to interfere with the organic constituents of the blood, nor imitate the animal extractives and protein compounds of the circulating fluid, in order to supply them if deficient. There is, however, no occasion for this in Asiatic cholera, for the evacuations from the intestinal surface, which destroy the healthy characters of the blood in that disease, appear to contain but little organic matter, being chiefly made up of water holding the salts of the blood in solution. Thus Vogel and Wittstock agree in describing cholera motions as containing intestinal mucus, traces of albumen, and the ordinary salts of the blood, with carbonate of soda somewhat in excess. The analysis of cholera blood again points clearly to the necessity of supplying more especially salts and water, if we desire to restore it to the healthy standard.

"With these facts before us, it is obvious that if we inject the veins at all, a solution of the salts of the blood is indicated in cases of Asiatic cholera: and it would seem well that those who may think it advisable to have occasional recourse to that plan of treatment for the recovery of severe and advanced cases, should keep a mixture of saline ingredients, combined in the proportions (or as nearly so as possible) observed in healthy blood. In order to answer the above requirements, I would recommend the following powder to be prepared, and kept ready for solution:—Chloride of sodium, three ounces; phosphate of soda, one ounce; carbonate of soda, one ounce and a half; sulphate of soda, half an ounce.

"As regards the physical peculiarities of the fluid for injection, it is quite as important that its characters in this respect should be attended to as that its chemical constitution should bear its proper relation to the blood. The specific gravity and temperature of the injection will require to be carefully adjusted before we can expect to put our patient under the conditions most favorable for recovery when mixing it with the blood. With regard to temperature but little need be said, 98° Fah. it is necessary, as being that of healthy blood; and I would only remark, that it should be determined by a correct thermometer, and not by one of those cheap instruments with which the market is just now unfortunately inundated. The specific gravity should be adjusted by gradually diluting a strong solution of the saline mixture above described with small portions of distilled water, at the temperature of from 58° to 63° Fah., occasionally testing it with the saccharometer (the ordinary instrument used in the examination of urine), until the instrument indicates a specific gravity of 1030. This being attained, the fluid has merely to be brought to the temperature of 98° Fah. to fit it for use."

In the London Lancet we find the following extract of a letter from Dr. Ayre in reference to the treatment of Cholera by minute doses of Calomel frequently repeated!

I shall here briefly repeat, for the sake of obviating all misconception on the subject, the leading particulars of what is denominated my plan of treatment, and which consists in the stage of collapse of giving one or two grains of calomel every five or ten minutes, with one or two drops of laudanum with the first few doses of the drug, and in perseveringly continuing the same dose at the same intervals of time, until the symptoms of collapse become materially subdued. This plan I have uninterruptedly pursued from the first to the last patient I ever attended in the disease, amounting to a very large number; and my reason for giving the small dose of the calomel was, that large ones were rejected from the stomach, and I repeated it frequently because it was small, and that thus the action of the medicine on the stomach might be constantly kept up in a disease whose duration is to be counted by minutes. I gave the minute dose of the opiate to enable the stomach to retain the calomel, and prevent its too early descent into the bowels, and not as a sedative. I abstained from the use of all auxiliary treatment in my early cases, that I might not compromise the conclusion at which I desired to arrive—as to the remedial power of calomel; and I have uniformly avoided them since, because I found that calomel in doses small and frequently repeated was the alone remedy. I have never given stimulants in any form, because I found them not to be necessary, and believed they would prove pernicious when, from the long duration of the collapse, and the delay in commencing the treatment, consecutive fever might be feared; and lastly, I fixed no limit to the quantity of calomel which I gave than that which the duration of the collapse prescribed, having become early assured that pending its continuance no absorption of the calomel into the system takes place, and that whilst it is so given, no salivation or other inconvenience is induced by it, and that no extremity to which a patient may be reduced can justify our withholding or abandoning the use of it.

A report was issued from the English board of health, April 17, 1854, from which it appears that the disease is more rapid in its course there than at any former visitation. The premonitory is shorter, or in many cases scarcely exists. The proportion of the better classes attacked is far greater than in previous epidemics. One fact is mentioned in that report which is of special importance, and which should be well understood, namely, that "some of the powerful predisposing causes of the disease cannot be removed during the actual presence of the epidemic, without the risk of increasing the evils intended to be remedied." In reference to this subject, they hold the following language:—"The board deem it necessary again to caution the local authorities against such a culpable mode of proceeding, which even in ordinary seasons would be attended with imminent danger; but that danger is greatly increased at an epidemic period."

Facts of the most convincing kind are adduced to show that where precautionary measures are resorted to in time, and executed with promptness and energy, the disease has been unable to gain a footing.

Dr. Mackintosh, in the London Lancet for April, 1854, recommends sugar in the treatment of Cholera. He gives two ounces of sugar in six ounces of camphor mixture, with a few drops of spirits: dose, a table spoonful every ten minutes. This is accompanied with beef-tea and wine.

A writer in the Medical Times and Gazette says that of all remedies heretofore tried as a palliative, with the exception perhaps of opium, there is none more deserving of a further trial than quinine.

The cold douche has been used by Dr. E. McPherson with much benefit. This writer does not consider the premonitory diarrhœa as a part of the disease.

In the French journals we find but little of interest. Its cause, mode of propagation, pathology, and treatment furnish still the fruitful source of numberless theories. In the meantime, it has steadily pursued its course, choosing its victims from all classes of society, and doing its work with a fearful rapidity. From one of the leading French journals we make a few translations.

In the *Gazette Medicale*, for April 15, we find the following ondensed report of a paper read before the Academy of Medi-ine, by Prof. Piorry, on the cases of epidemic cholera which ave appeared in the wards St. Charles and St. Anne of the La harite, 1854.

The facts contained in this article have conducted M Piorry to the following conclusions in reference to the causes, symptoms, diagnosis pathology, and treatment of this disease

Everyt ing leads us to believe, says he, that the immediate cause of this disease is a special virus, which has not yet received a name, but which, on account of its Indian origin and its terrible effects, should be called *indoliose* (the virus of the Indian pest). Some facts of this last visitation of the epidemic go to show that cholera may in certain conditions and certain localities be communicable.

Another important point to which M. Piorry has called attention, is that in November, 1853, and especially in March last, there was in the city only a small number of persons affected by the epidemic, that in the general hospitals there were but a few cases, while La Charite was specially smitten. The wards St Charles twice and St. Anne once contained a very large number of cholera patients in proportion to those in the other departments of the house But these two wards are the most poorly ventilated of any in the establishment; in addition, the privies and their sewers have constantly emitted a fœtid odor which it has been impossible to prevent or destroy. These facts, according to M. Piorry, are in accordance with those observed at La Salpetriere and elsewhere in 1832 relative to the influence of confinement and alteration of the air upon the appearance, development and severity of cholera.

M Piorry has also observed that in Nov., 1853, and March, 1854, as in 1849, the prevailing winds were from Siberia towards the south-west, while the cases that seemed to bear a relation to each other, as from contagion, presented the symptoms rather of cholerine than of cholera asphyxia.

M. Piorry and M. Blair, his chef de clinique, in imitation o M Briquet. has had recourse to injections into the veins This operation in four cases for a short time produced a mark d improvement of all the symptoms in patients already reduced to the last extremity The pulse returned; the expression of the features became better; the energy was increased, etc.; but after a few hours the improvement ceased, the diarrhœa returned, and the collapse was again established with the same intensity.

M. Piorry has asked whether it may not be possible by repeating several times a day these injections, to restore to the blood the water which it has lost. This operation has not been followed by any accident. He has attempted to make his patients inspire the vapor of water; this has not succeeded. Seeing at length that neither drinks nor injections, nor the vapor of water inhaled, was attended with success he has tried injections of water into the bladder, always with a view to the absorption of the water. He has thought that this means has been followed by an amelioration of the symptoms in one case.

In the same journal for April 22, Dr. G. F. Grarich, physician at Beyrout, recommends an infusion of garlic internally, and cataplasms of the same externally. The success attending this remedy in his hands renders it worthy of further trial.

In an editorial of the same journal, for April 29, after speaking of the spread of Cholera through the city of Paris and the surrounding country, the writer goes on to remark that there is a notable difference between the cholera as then existing and that which occurred at the end of the year 1853. This difference relates principally to the duration and degree of the cold stage, the intensity of the cyanosis, and the general character of the cramps; that many light cases of cholera convalesce without presenting a distinct period of reaction, and that in the larger proportion of severe cases, seven out of eight, reaction was not established, even when the patient lived till the end of the second or third day, and that the intensity and duration of the cold stage bears a marked relation to the epidemic development of the disease. In this respect it resembles more the cholera of 1817, 1828, and 1832.

In regard to the diarrhœas which precede, accompany and follow the epidemic, the author seems to consider them as bearing an important relation to true cholera. The lighter cases in which, the stools are semi-liquid, without rumbling and without crepitation, usually accompany the epidemic in its mildest form, while the graver cases, with liquid stools, eructations, a patulous feel to the abdomen, with contraction of the abdominal wells, indicates the existence or rapid approach of true cholera. These diarrhœas become more and more severe until, according to our author, it is impossible to say where what may appropriately be called the petit mal ends and the grand mal commences This writer believes in the contagiousness of the disease, and thinks it impossible to see any difference as to the mode of its propagation between cholera and variola, rubiola and scarlatina.

It will be seen from the foregoing extracts, that but little that is really new has been published, either in England or on the Continent. The treatment is perhaps more simple than it has been in previous visitations. One of the errors both of the people and the profession has been too much medication; agents of the most active kind have been used in the greatest profusion and without any intermission. It is not probable that any specific will be discovered; but the principles governing its treatment furnish a legitimate subject of inquiry.

We may remark in reference to injections into the veins, that we have tried it in one case. The patient was in complete collapse, perfectly pulseless; after a few ounces had been injected the pulse returned, and there was a temporary amelioration of all the symptoms; but our hopes were disappointed. We should not hesitate however to repeat it under similar circumstances.

Chicago, July 1854.

